# Complications following Iliac Wing Fibrosarcoma

**DOI:** 10.1155/2019/9259571

**Published:** 2019-11-30

**Authors:** G. Stan, H. Orban, R. Deculescu

**Affiliations:** ^1^UMF Carol Davila, Bucharest, Romania; ^2^Orthopaedics and Traumatology, Elias Universitary Hospital, Bucharest, Romania

## Abstract

The aim of this case report is to underline surgical strategies for complications in a case of a young man with fibrosarcoma of the bone treated with pelvic resection followed by reconstruction with massive bone allograft. A type I pelvic resection was performed as a radical resection of tumor followed by a biological reconstruction of iliac wing using frozen allograft. The iliac allograft was fixed in place using 4 screws. The immediate postoperative period was complicated with local sepsis of reconstructed site treated with pediculate omentoplasty. After 1 year from surgery, the X-ray exam showed an integrated allograft. After 20 years from the first surgery, the patient presented with the left hip pain of 3-month duration with mechanical pattern. The X-ray and CT exam showed the left hip arthritis and no signs of recurrence. A total hip arthroplasty with dual mobility cup and uncemented stem was performed. Despite the immediate postoperative local infection, the allograft was left in place and integrated after all. Omentoplasty could be a very useful technique in eradicating local infection, due to the immunogenic properties of the omentum. The allograft is still strong enough to give support for a hip arthroplasty at 20 years after implantation.

## 1. Introduction

The prognosis and survival in primary pelvic bone tumors are less favorable than in other locations due to the complexity of anatomy of the region and secondary to the difficulty of radical resection surgery, meaning an increased rate of recurrence [1]. The goal of every tumoral resection surgery is to achieve free-of-disease edges of bone resection. The surgical treatment includes hemipelvic resection and limb salvage procedure with or without postoperative reconstruction [2–4]. Limb salvage procedures followed by reconstruction are more commonly used than hemipelvic resection due to the fact that patients have a better functional prognosis. The 5-year survival rate is not statistically different between reconstructive surgery with adjuvant therapy and nonreconstructive amputation [5, 6]. Fibrosarcoma of the bone represents 5% of the primary malignant tumors of the bone. The peek age incidence is around 35-45 years with equal distribution between men and woman. It is mainly localized around the knee joint [7]. The aim of this study is to underline surgical strategies for complications in a case of a young man with fibrosarcoma of the bone treated with pelvic resection followed by reconstruction with massive bone allograft.

## 2. Case Report

**Figure 1:**
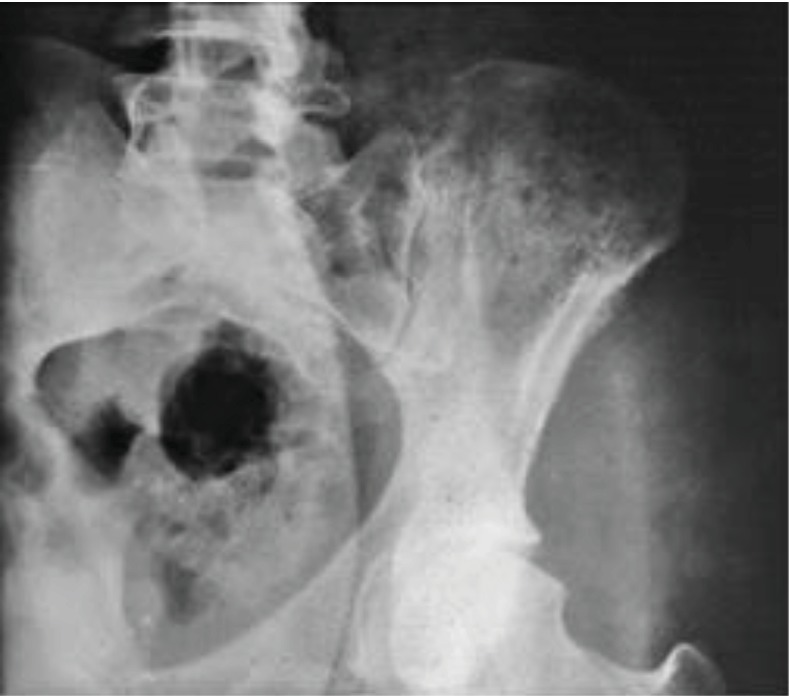
Iliac crest fibrosarcoma X-ray.

A 24-year-old man presented himself with the left hip pain with nocturnal exacerbation and difficulty in walking of 6-month duration. On physical exam, a firm painful mass was palpable in the posterior part of the left iliac wing. The tumor was fixed in relation to the adjacent structures. Also, the pain was exacerbated by the motion and prolonged orthostatism. Laboratory testing showed elevated levels of erythrocyte sedimentation rate and alkaline phosphatase. The lung X-ray exam was normal. The pelvis X-ray showed a highly destructive osteolytic zone in iliac crest above the acetabulum, close to the sacroiliac joint (Figure 1). The CT scan showed the same lytic lesion measuring 5/3.8/4 cm with no invasion in periarticular areas. The decision was to perform first an open biopsy of tumoral mass. The result confirms that the tumor was a high-grade fibrosarcoma of the iliac bone. Based on the biopsy result and imaging testing, the lesion was staged as II B sarcoma, according to the Enneking classification of bone tumor.

**Figure 2:**
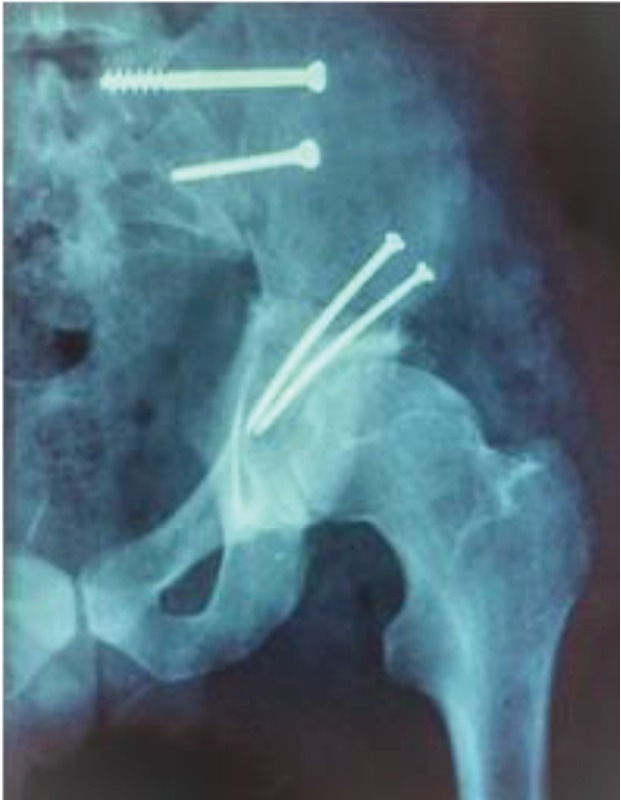
Allograft reconstruction.

Next, a type I pelvic resection was performed as a radical resection of tumor followed by a biological reconstruction of iliac wing using frozen allograft. The iliac allograft was fixed in place using 4 screws (Figure 2).

**Figure 3:**
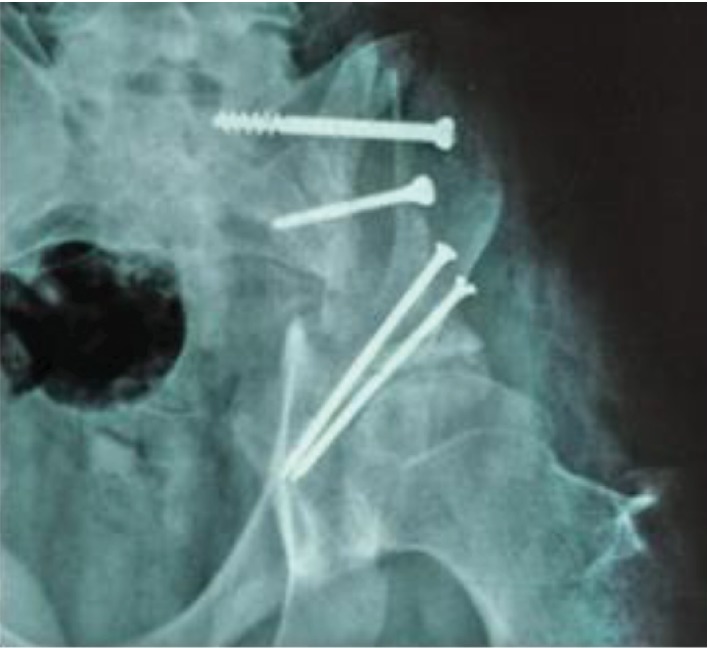
Resorption of the graft. 1 year from reconstruction.

Adjuvant chemotherapy was needed in 6 stages using doxolem, cyclofosfamide, and methotrexate. No neoadjuvant chemotherapy was used. The immediate postoperative period was complicated with local sepsis of reconstructed site treated with pediculate omentoplasty. After 1 year from surgery, the X-ray exam showed an integrated allograft. The walking was possible without crutches, and the patient returned to normal activity. The partial resorption of the graft was observed in the unloaded area (Figure 3). Still, the pelvic ring was closed.

**Figure 4:**
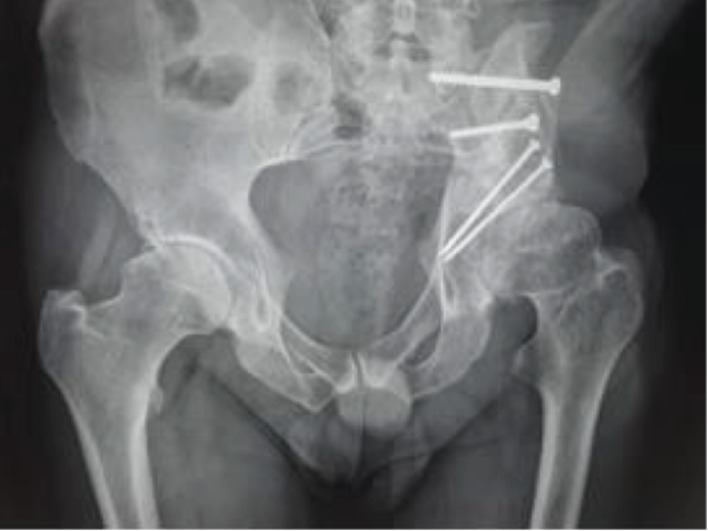
20 years from reconstruction. Hip arthritis.

**Figure 5:**
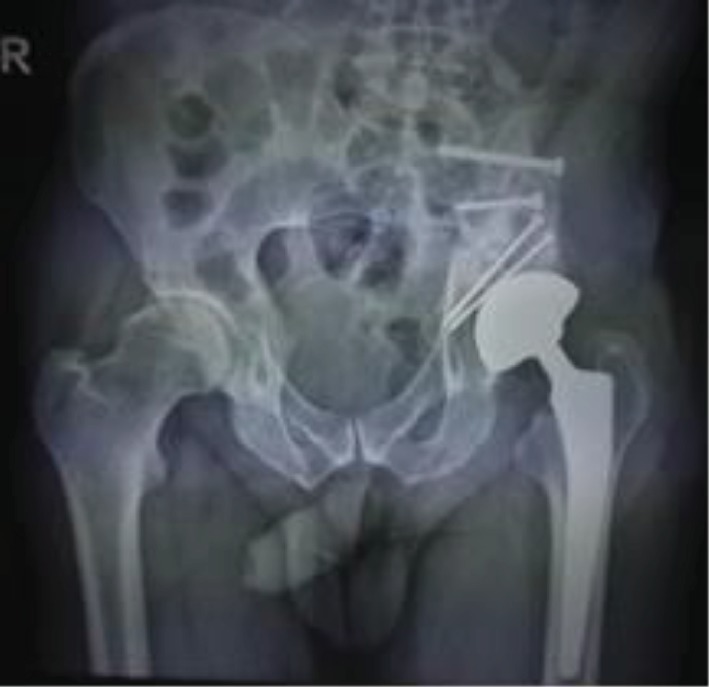
Hip arthroplasty—3 months after surgery.

The hip was free of pain, and a shortening of 2 cm was assessed. The imaging studies showed no recurrence of the disease. After 20 years from the first surgery, the patient presented with the left hip pain of 3-month duration with mechanical pattern. The X-ray and CT exam showed the left hip arthritis (Figure 4) and no signs of recurrence. On physical exam, a shortening of 2 cm was observed. Trendelenburg sign was positive on the left side, and quadriceps amyotrophy was noted. The Harris Hip Score was 64. The inflammatory tests were negative, and no punction was performed before surgery for excluding infection. A total hip arthroplasty with dual mobility cup and uncemented stem was performed, using an anterolateral approach (Figure 5). Samples of tissue were taken during surgery for bacteriological tests. The lab tests for infection were negative. The rehabilitation program started the next day after surgery, with active exercise and full weight bearing on the left side with the help of crutches.

## 3. Discussion

The usual complications after pelvic resections are intraoperative bleeding, infection, nerve injuries, ureter, bladder, and bowel injuries; wound-healing complications, dislocation of prostheses, allografts resorption, lower quadrant hernia, bowel ischemia, and late venous thrombosis [8]. Increased rates of infection in reconstruction procedures and mechanical complications are due to the laborious surgical procedures and the poorly vascularized residual region. Reconstructions using pelvic prostheses have been associated with complication rates as high as 60%, with 40% of patients needing repeat surgeries [9, 10]. Data from literature show five-year survival rates ranging from 40% to 70% for surgically treated patients according to histological grade and tumor volume as prognostic factors [11]. Bergh et al. have also suggested age as a prognostic factor [12]. Our patient was suffering from an aggressive tumor, stage II B, with a volume of 5/3.8/4 cm. The resection-reconstruction surgery was performed at the age of 24 years. Although aggressive and bulky, the tumor was completely removed thanks to the oncological resection in a fashion that permits reconstruction of the pelvic wing and returning to normal activity of the young patient. At 20 years from resection, there was no sign of recurrence. Infection may be associated with large and extensive approaches resulting in large cavities. Literature showed that reconstruction is the only independent significant prognostic factor of infection after pelvic tumor resection [13, 14]. The resulting large dead space after resection, foreign material, and prolonged surgical time are all arguments for the high risk of infection, particularly after pelvic reconstruction. In our case, the patient developed local sepsis at 3 weeks postoperatively. The decision was to fill up the death space with the help of omentoplasty. The omentum has very good hemostatic qualities due to a combination of tamponade and biochemical factors. Thromboplastin is also high concentrated in the omentum which is believed to have a critical function in hemostasis and thrombogenesis. Reduction of dead space by highly vascularized tissue, the omentum, has the property of angiogenesis and capillary ingrowth. These qualities of the omentum make it suitable as a graft in pelvic exenterations [15]. The greater omentum because of its immunogenic properties is particularly useful, offering excellent results in the control of infection [16]. A high rate of local complications must be anticipated after using reconstruction with massive bone allograft especially after tumoral resection. In his study, about local complications after allograft reconstruction, Delloye has found the first three major ones to be nonunion, fracture, and infection [17]. According to Mankin [18], the highest frequency of infection occurred in patients with tumor surgeries and most of the infected grafts failed. Because of using omentoplasty in our case, the infection was eradicated and the allograft was left in place and was integrated after all. Breakage or partial resorption after large allogeneic bone transplantation is a common complication after reconstruction [6]. In our case, we observed partial allograft resorption in the unloaded area after 3 years. Despite this, the pelvic ring continuity was maintained. Also, 2 cm of shortening of the left side and a minor pubic disjunction were noted. The resulting muscular imbalance and unusual loads occurring on the left hip contribute to the appearance of hip arthritis 20 years later. Also, the acetabular coverage of the femoral head was diminished after resection, thus contributing to arthritis. The treatment indication was represented by the total hip arthroplasty. The major concerns of this procedure are the stability of the implant, due to the muscular insufficiency resulted after previous surgery, and the integrated allograft support for the implant. Intraoperatively, a strong bone structure was noted and uncemented cup implantation was possible without using a additional graft support or augmentation. The surgeon decided to use an uncemented total hip implant with dual mobility cup. The dual mobility cup allows a reduction in the dislocation rate without compromising clinical outcomes and implant longevity. The dual mobility component increases hip range of motion until impingement occurs through its two-articulation design. If the femoral neck and the rim of the PE liner come into contact, a second articulation begins to function and consists of the back of the PE liner and the metallic acetabular shell [19]. Several studies have reported a low rate of postoperative implant instability [20, 21]. The rehabilitation protocol started the first day after surgery, and the full weight bearing was allowed with the help of crutches. At 6 weeks of follow-up, Harris Hip Score was improved from 64 to 95. No complications were noted so far.

## 4. Conclusion

Pelvic bone tumors remain a great challenge for surgeon in terms of oncological resection and pelvic reconstruction. Despite the immediate postoperative local infection, the allograft was left in place and integrated after all. Omentoplasty could be a very useful technique in eradicating local infection, due to the immunogenic properties of the omentum. The allograft is still strong enough to give support for a hip arthroplasty at 20 years after implantation. Even if tumoral disease is cured, major complications are still possible any time after the first surgery of resection reconstruction, meaning that a close follow-up is necessary.
